# In vivo superresolution photoacoustic computed tomography by localization of single dyed droplets

**DOI:** 10.1038/s41377-019-0147-9

**Published:** 2019-04-03

**Authors:** Pengfei Zhang, Lei Li, Li Lin, Junhui Shi, Lihong V. Wang

**Affiliations:** 1 Optical Imaging Laboratory, Department of Biomedical Engineering, Washington University in St. Louis, St. Louis, Missouri 63130 USA; 2 Caltech Optical Imaging Laboratory, Andrew and Peggy Cherng Department of Medical Engineering, Department of Electrical Engineering, California Institute of Technology, 1200 E. California Blvd., MC 138-78, Pasadena, CA 91125 USA; 3 Present Address: School of Precision Instruments and Optoelectronics Engineering, Tianjin University, Tianjin, 300072 China

**Keywords:** Photoacoustics, Imaging and sensing

## Abstract

Photoacoustic (PA) computed tomography (PACT) is a noninvasive hybrid imaging technique that combines optical excitation and acoustic detection to realize high contrast, high resolution, and deep penetration in biological tissues. However, the spatial resolution of PACT is limited by acoustic diffraction. Here, we report in vivo superresolution PACT, which breaks the acoustic diffraction limit by localizing the centers of single dyed droplets that are flowing in blood vessels. The droplets were prepared by dissolving hydrophobic absorbing dye in oil, followed by mixing with water. The dyed droplets generate much higher-amplitude PA signals than blood and can flow smoothly in vessels; thus, they are excellent tracers for localization-based superresolution imaging. The in vivo resolution enhancement was demonstrated by continuously imaging the cortical layer of a mouse brain during droplet injection. The droplets that were flowing in the vessels were localized, and their center positions were used to construct a superresolution image that exhibits sharper features and more finely resolved vascular details. An improvement in spatial resolution by a factor of 6 has been realized in vivo by the droplet localization technique.

## Dyed droplets improve photoacoustic imaging

Dyed droplets improve the resolution of an imaging technique involving laser pulses and ultrasound. Lihong V. Wang of the California Institute of Technology and colleagues demonstrated improved resolution of ‘photoacoustic computed tomography’ (PACT) by a factor of six by injecting dyed droplets into brain blood vessels of live mice. In PACT, tissues receiving short bursts of laser pulses emit ultrasound waves that are detected by an array of sensors. The technique is used for high-spatiotemporal-resolution imaging of biological tissues with super-contrast and deep penetration. The injected dyed droplets acted as a good contrast medium, producing much higher ‘photoacoustic’ signals than the blood background, while also flowing smoothly through the blood vessels. By localizing the centers of the dyed droplets, a higher resolution image can be formed. Further improvements, by using dyed droplets with higher optical absorption, for example, could lead to applications in blood capillary imaging and in monitoring targeted drug delivery.

## Introduction

Photoacoustic tomography (PAT) is a nonionizing, hybrid imaging modality that combines optical excitation and acoustic detection to realize high optical contrast and high spatial resolution at depths inside biological tissues^1,2^. In PAT, nanosecond-pulsed laser illumination of absorbing molecules generates volume expansion due to the transient local temperature rise. The rapid thermoelastic expansion of the stressed tissue generates acoustic waves that propagate in the tissue—with orders of magnitude weaker scattering than light scattering on a per-unit-path-length basis in the acoustic frequency of interest—and are detected by an ultrasonic transducer or a transducer array^3,4^. PAT inherits the advantage of high optical contrast in optical imaging methods while breaking the optical diffusion limit on the penetration of high-resolution optical imaging by detecting ultrasound. PAT can operate in both the optical ballistic and diffusive regimes, thereby providing multiscale imaging solutions. By scanning a tightly focused laser beam in the optical ballistic regime, a PAT system can realize optical-resolution photoacoustic microscopy (OR-PAM)^5,6^. Recently, OR-PAM has been used for high-speed, high-resolution mapping of the cortical blood vessel network and to study the hemodynamics in the mouse brain cortex^7^. The penetration depth of OR-PAM is typically limited to ~ 1–2 mm because its high spatial resolution depends on the tight optical focus of ballistic photons. Photoacoustic computed tomography (PACT) detects acoustic waves that are generated by both ballistic and diffused photons and retrieves the optical absorption distribution through an inverse algorithm, allowing for high-resolution imaging at a depth of up to several centimeters^1^. PACT has been used for imaging small-animal whole-body dynamics, functional whole mouse brain hemodynamics, and human breast tumors at high spatiotemporal resolutions^8–10^.

However, the resolution of PACT is fundamentally limited by acoustic diffraction and, thus, by the acoustic wavelength in tissues. Although finer resolution can be realized by detecting higher frequency ultrasound, the associated increase in ultrasound attenuation decreases the penetration depth^1,2^. Inspired by superresolution fluorescence imaging techniques, such as photoactivation localization microscopy (PALM) and stochastic optical reconstruction microscopy (STORM)^11,12^, several techniques have been used to break the acoustic diffraction limit in PACT. One such technique utilized the PA signal fluctuations that were induced by either speckle illumination or flowing absorbers^13,14^. More recently, superresolution PA imaging has also been demonstrated by the localization of flowing microbeads^15,16^. However, none of these techniques have been successfully applied to in vivo imaging. The main disadvantage of the fluctuation-based technique is that the speckle contrast becomes too low for detection in deep tissues due to the orders of magnitude smaller size of the fully developed speckle grains compared to the detection acoustic wavelength. Moreover, one of the main drawbacks of the bead-based localization technique is that solid beads can jam small blood vessels and block blood flow, thereby impeding in vivo applications.

In this paper, we report a novel technique for in vivo superresolution PACT. This technique breaks the acoustic diffraction limit by localizing flowing dyed droplets. The droplets were prepared by dissolving hydrophobic dye IR-780 iodide in oil, followed by mixing with water. The dyed droplets produce much higher-amplitude PA signals than the blood background and can flow smoothly in blood vessels. Once injected into the bloodstream, the droplets can be tracked and localized with high precision due to their high absorption contrast. The in vivo resolution improvement was demonstrated by imaging the cortical layer of a mouse brain during droplet injection. A superresolution image that was constructed via localization of ~ 220,000 droplets showed sharper features and resolved finer vascular details. Single droplets were also tracked in the deep brain, and their flow directions and speeds were characterized. The experimental results suggest that the proposed technique is a promising tool for imaging blood capillaries in deep tissues.

## Results

### In vitro characterization of the droplets using PACT

**Fig. 1:**
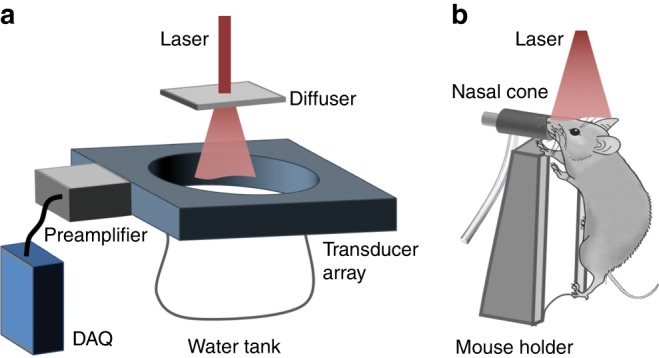
Schematic diagram of the superresolution PACT setup for mouse brain imaging. **a** PACT system, which is equipped with a full-ring ultrasonic transducer array. **b** The horizontal plane of the mouse brain was aligned parallel to the transducer array focal plane for cortical imaging

**Fig. 2:**
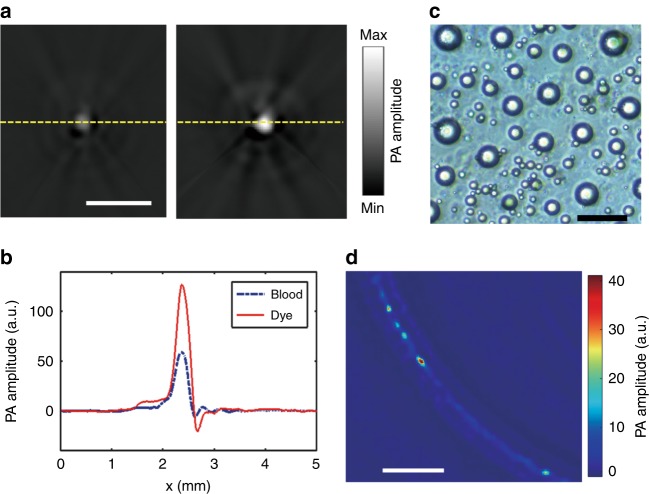
Infrared dye IR-780 iodide as an optical contrast agent for superresolution PACT. **a** PACT images of whole bovine blood (left) and dye solution (right) in a capillary tube. The dye solution (2 mM) was diluted by a factor of 100. **b** PA amplitude profiles along the dashed lines in **a**. **c** A photograph of the dyed droplets suspended in distilled water. **d** A PACT image of a silicone capillary tube that was filled with bovine whole blood and dyed droplets. The scale bars are 2 mm, 50 µm and 2 mm in **a**, **c**, **d**, respectively

The droplets were prepared by mixing oil-dissolved dye (IR-780) with water (“Materials and Methods” section). The molar absorption coefficient of the IR-780 iodide dye solution is 300 times higher than that of hemoglobin (80% oxy-hemoglobin and 20% deoxy-hemoglobin) at 780 nm (Supplementary Fig. S1). Based on the normal hemoglobin concentration (150 g L^−1^) in whole blood and the gram-molecular weight of hemoglobin (64,500 g mole^−1^)^17^, the absorption coefficient of the dye solution at 2 mM is approximately 260 times higher than that of whole blood. To compare the PA signal of the dye solution with that of whole blood, we diluted the dye solution (2 mM) with the oil mixture to be described subsequently by a factor of 100 and injected it into a silicone capillary tube of 300 µm in inner diameter. Then, the tube was sealed at both ends and imaged by PACT (Fig. 1a, “Materials and Methods” section). The tube was placed perpendicular to the full-ring ultrasonic transducer array’s imaging plane such that it appeared as a single disk in the reconstructed image, as shown in Fig. 2a, right. The same procedure was performed with whole bovine blood (Innovative Research), and the reconstructed image is shown in Fig. 2a, left. When excited at 780 nm, the dye solution produced a PA signal that was 2 times higher in amplitude compared to the blood, even after the dye solution was diluted by a factor of 100. The PA amplitude profiles along the dashed lines in Fig. 2a are plotted in Fig. 2b, according to which the PA amplitude of the original dye solution was approximately 200 times higher than that of the whole blood. These results demonstrated that this dye is a good candidate for PACT as a contrast agent in blood vessels. Then, the dye solution (2 mM) was used to produce droplets by mixing with water, as detailed in “Materials and Methods” section. The sizes of the droplets that were prepared via this approach are dispersed and range from 4 to 30 µm, with a mean value of approximately 10 µm, as shown in Fig. 2c. The small sizes and liquid compliance of the droplets guarantee that they can flow smoothly in blood capillaries, while a single droplet can generate PA signals of sufficiently high amplitude relative to the blood background.

Due to the high contrast, the droplets in the bloodstream are effective tracers. A silicone capillary tube with a 300-µm inner diameter was embedded in 3% agar gel to mimic a blood vessel; we injected whole bovine blood that was mixed with the droplet suspension with a ratio of 1000:1 into the tube. The concentration of the droplets was controlled so that, statistically, no more than one droplet appeared in an acoustic diffraction-limited volume at any time. Then, the phantom was imaged by PACT with the capillary tube lying in the focal plane of the transducer array. Figure 2d shows a PACT image of the capillary tube, where the negative amplitudes have been removed. Even when surrounded by blood, single droplets can be identified. Once injected into the bloodstream, the droplets, which are flowing through the blood vessels, are tracked by PACT (Supplementary Movie 1) to visualize the vascular network. Single droplets are localized with high precision, and their center positions are used to construct an image with a spatial resolution that breaks the acoustic diffraction limit (“Materials and Methods” section, Supplementary Fig. S2)—a strategy that is widely used in single-molecule localization-based superresolution fluorescence microscopy and single-microbubble localization-based superresolution ultrasound imaging^18^.

### In vivo PACT of dyed droplets

In the in vivo study, droplets were used to improve the spatial resolution in PACT of a mouse brain vasculature. To image the cortical layer, the mouse was mounted onto a holder with its cortical plane oriented horizontally, as shown in Fig. 1b. The mouse holder was equipped with a Peltier heating plate and a thermocouple for temperature regulation. Then, the mouse was placed under the water bag of the imaging system, and ultrasound gel was applied between the head and the water bag for ultrasonic coupling. The holder was lifted until the brain’s cortical layer was in the focal plane of the transducer array. The expanded laser beam was incident downwards on the brain to excite PA waves.

**Fig. 3:**
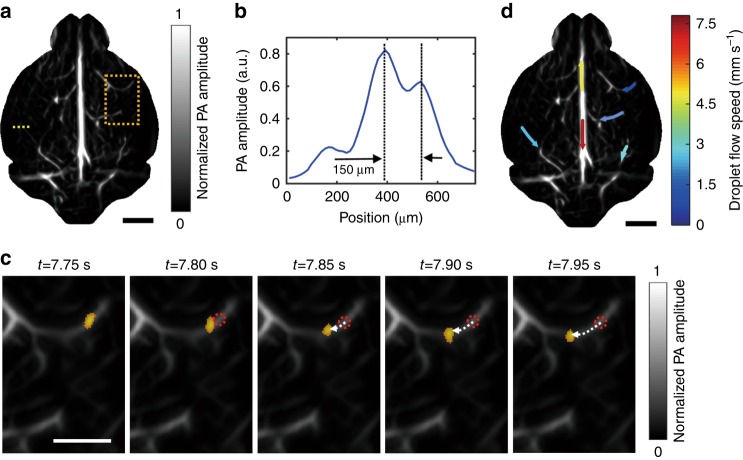
Tracking of droplets in the brain in vivo. **a** A unipolar image of the baseline cortical vasculature that was acquired prior to the injection of droplets, where a Hessian-based Frangi vesselness filter was applied. **b** A profile of the PA amplitude along the dashed line in **a**. **c** The flow of a droplet (orange dot) in brain vessels, which was tracked over time. The droplet images were overlaid on the baseline vascular image in the region that is bounded by the dashed rectangle in **a**. The dotted red circles indicate the initial locations of the droplets, and the dashed arrows indicate the flow pathway and direction. **d** The droplet flow velocities for several cortical vessels. The colors indicate the flow speeds, and the arrows indicate the flow directions. The scale bars are 2, 1 and 2 mm in **a**, **c**, **d**, respectively

The mouse brain was initially imaged using hemoglobin as the endogenous optical contrast. The laser was tuned to 780 nm, and the pulsed energy was set to 100 mJ. The optical fluence on the head surface was 30 mJ cm^−2^. The reconstructed bipolar image (Supplementary Fig. S3) was converted into a unipolar image by applying a Hessian-based Frangi vesselness filter to enhance the contrast, as shown in Fig. 3a. To quantify the spatial resolution, we plotted the profile of the PA amplitude along the dashed line, where a small vessel began to bifurcate into two. The distance between the two peaks in Fig. 3b corresponds to a spatial resolution of approximately 150 µm. Then, the brain was continuously imaged, while droplets were injected into the heart through the catheter. As discussed above, the droplet suspension was prepared with 20 μL of the dye solution (2 mM) in 1 mL of water, thereby leading to a droplet concentration of approximately 4 × 10^7^ mL^−1^. A 1-mL syringe that was filled with the suspension was connected to the catheter by a 25 G needle, and the suspension was injected by a syringe pump (EW-74905-52, Cole-Parmer). Due to the presence of organic molecules, such as lipids, in the blood, the droplets might be eventually dissolved by the blood after multiple circulations. In this way, the dyed droplets can be cleared out by the body. In addition, droplets could split into smaller ones during the pulmonary circulation and became undetectable. These factors limited the circulation time of the detectable droplets in vivo to ~ 8 min. To maintain the droplet concentration in vivo, a 6-s infusion at a pumping speed of 50 μL min^-1^ was repeated every 4 min until the end of the imaging session. The droplet concentration in the blood was estimated to be 1 × 10^5^ mL^−1^ based on the volume of the droplet suspension during each injection cycle (5 μL) and the blood volume in a mouse ( ~ 1.5 mL^19^). Thus, the number of droplets that appeared in an acoustic diffraction-limited volume (150 μm × 150 μm × 500 μm) was approximately 1.25. However, because the PA amplitudes of some small droplets were within the fluctuations of the blood background, the average number of droplets that were detectable in an acoustic diffraction-limited volume was less than one, which enabled the tracking and localization of single droplets in the mouse brain. The PACT system began to acquire data approximately 10 s after the initial injection, when the injected droplets had been pumped into the brain by the heart. The cortical layer was continuously imaged for half an hour with a frame rate of 20 Hz, thereby resulting in a total of 36,000 frames. During the data acquisition process, the mouse was anesthetized by 1% isoflurane at an air flow rate of 1 L min^-1^. The universal back-projection algorithm^20^ was used to convert the radio-frequency (RF) data into images.

As shown in Supplementary Movie 2, numerous single droplets flowed smoothly in the vessels, which are indicated by the bright migrating spots. Although large droplets became momentarily trapped in the small vessels, they were cleared quickly by blood flow, thereby taking advantage of the liquid characteristics of the droplets. The reconstructed images were denoised and subtracted from the adjacent frames to highlight flowing single droplets, as detailed in “Materials and Methods” section. Figure 3c shows a droplet that is flowing in a vessel over time. The droplet flow direction and speed were quantified from the time-lapse images, as detailed in “Materials and Methods” section. Due to the high droplet flow speed and the relatively low imaging frame rate (20 Hz), droplets sometimes traveled through a vessel in only a few imaging time points. Therefore, the spatial resolution of the flow speed mapping is low. As shown in Fig. 3d, the droplet flow speed in the cortex was 1.3–7.5 mm s^-1^, which well accords with the previously reported blood flow speed in the cortex^21,22^. The flow speed that was determined in this way was extracted from multiple droplets; some droplets flowed even faster and reached 26 mm s^-1^ in some vessels. Interestingly, the droplets in the upper and lower parts of the superior sagittal sinus (SSS) flowed in opposite directions; a possible reason is that vessels from different depths that had different flow directions overlapped with each other.

**Fig. 4:**
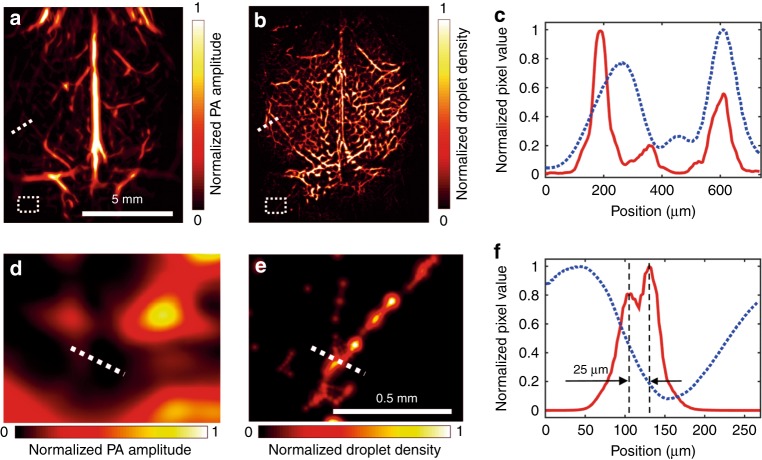
In vivo imaging of the cortical layer of a mouse brain. **a** A unipolar image of the cortical layer that was acquired by conventional PACT using hemoglobin as the contrast, where a Hessian-based Frangi vesselness filter was applied. **b** A superresolution PACT image of the cortical layer by localizing single droplets; the same vesselness filter was applied as in **a**. **c** Amplitude profiles of the conventional image (blue dashed line) and the superresolution image (red solid line) along the dotted lines that are shown in **a**, **b**. **d**, **e** Magnified images of the regions that are bounded by dotted rectangles in **a**, **b**. **f** Amplitude profiles along the dotted lines in **d**, **e**, where the red solid line corresponds to the profile from the superresolution image, the blue dashed line corresponds to the profile from the conventional image, and the vertical black dashed lines indicate the locations of the two vessels

Then, the extracted images of single droplets were analyzed by 2-D Gaussian fitting, and their centers were identified. The localization precision depended on the contrast-to-noise (CNR) ratio of each droplet. According to a histogram of the droplets’ CNRs, most of the droplets exhibited CNRs of approximately 5, and the localization precision was estimated to be 10 μm by numerical simulation (Supplementary Fig. S4). In total, approximately 220,000 droplets were localized during the half-hour data acquisition, and their centers were used to construct a superresolution image, as detailed in “Materials and Methods” section and Supplementary Movie 3. Due to the limited total number of frames, the number of localized droplets in various regions might not be sufficiently high for connecting these spots into vascular features, thereby resulting in isolated spots (Supplementary Fig. S5). These isolated spots were removed by applying a Hessian-based Frangi vesselness filter using the same parameters as in Fig. 3a. Figure 4a, b show the images of the cortical-layer vessels that were obtained via conventional PACT and superresolution PACT, respectively. Compared with Fig. 4a, b shows substantial improvements in spatial resolution. The amplitude profiles of Fig. 4a, b along the dotted lines are shown in Fig. 4c. In the superresolution image, the vessels appear sharper, and the closely neighboring vessels can be resolved. The droplet-localization technique not only improves the PACT resolution but also lights up the vessels that are otherwise obscured by the blood background. Figure 4d, e show the magnified images of the regions that are bounded by dotted rectangles in Fig. 4a, b, respectively. While no features can be readily identified in the conventional image (Fig. 4d), the superresolution image displays bifurcated vessels (Fig. 4e). The amplitude profiles along the dotted lines are shown in Fig. 4f, according to which the two vessels, which are separated by 25 µm, can be resolved by the droplet-localization technique. These results suggest that the spatial resolution of PACT has been improved by a factor of 6 by the localization of single droplets.

A few features that appear in the conventional image do not appear in the superresolution image. The differences in the vascular network between the superresolution image and the conventional image are shown in the overlaid image (Supplementary Fig. S6). The artifacts were caused by the limited number of droplets in various vessels and because several features in the conventional image were not blood vessels. Moreover, the SSS shows a hollow feature rather than a filled line with a Gaussian-distributed cross-section, as in the conventional image. A possible explanation for this artifact is that dyed droplets flow faster along the axis of a large vessel and slower near the vascular wall^23^. Due to the low frame rate, the slower-moving droplets were easier to track, thereby resulting in more localized droplets near the vascular wall in the superresolution image. These artifacts can potentially be mitigated by increasing the frame rate in the future.

**Fig. 5:**
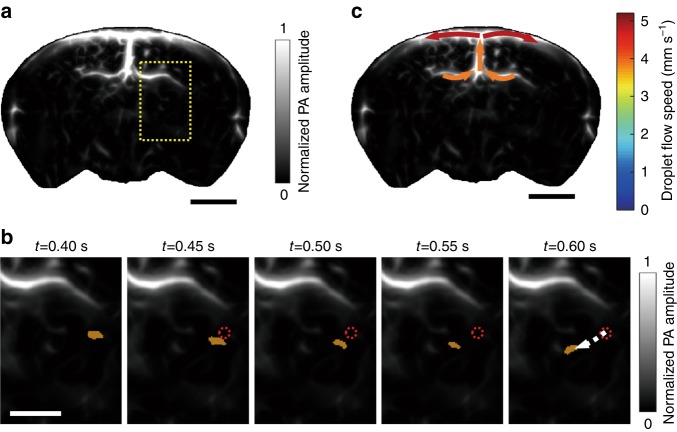
Tracking of droplets in the deep brain by PACT. **a** A unipolar image of the baseline vasculature in the coronal plane (Bregma –1.0 mm) that was acquired prior to the injection of droplets, where a Hessian-based Frangi vesselness filter was applied. **b** The flow of a droplet (orange dot) in the brain, which was imaged over time. The droplet images were overlaid on the baseline vascular image in the region that is bounded by the dashed rectangle in **a**. The dotted red circle indicates the initial location of the droplet, and the dashed arrow indicates the flow pathway and direction. **c** Droplet flow directions and speeds for several vessels. The colors indicate the speeds, and the arrows indicate the flow directions. The scale bars are 2, 1 and 2 mm in **a**-**c**, respectively

We also tracked single droplets in the deep brain by imaging the coronal plane. In this case, the mouse was held vertically with the tooth bar, which positioned the brain cortex perpendicular to the imaging plane of the transducer array. Then, the mouse was submerged into the water tank with its nose and mouth above the water, and the laser beam illuminated obliquely on the brain. We imaged the coronal plane (Bregma –1.0 mm) prior to the droplet injection. The reconstructed bipolar image (Supplementary Fig. S7) was converted into a unipolar image by applying the Hessian-based Frangi vesselness filter, as shown in Fig. 5a. After the droplet injection, we acquired 1200 PACT images continuously with a frame rate of 20 Hz. As shown in Supplementary Movie 4, single droplets were tracked in the deep brain. Figure 5b shows a droplet that is migrating at a depth of ~ 4 mm in the brain, and the flow speed of this droplet was determined to be 4.6 mm s^-1^. The droplet flow directions and speeds in several vessels in the coronal plane were also calculated, as shown in Fig. 5c. Due to the attenuation of both light and ultrasound when propagating inside the brain, the PA signals of small droplets in the deep brain were too weak to detect, while large droplets remained observable in the brain at a depth of 4 mm. However, the number of observable droplets in the coronal plane was insufficient for constructing a superresolution image in this work. This problem can potentially be overcome by using photoacoustically brighter dyes in the future.

## Discussion

In summary, we reported a technique for superresolution PACT in vivo. The technique realized 25-µm resolution in a live mouse brain, thereby breaking the acoustic diffraction limit by the localization of single flowing droplets in vessels. The droplets were prepared by dissolving hydrophobic dye IR-780 iodide in oil, followed by mixing with water. The dye is highly absorbing at 780 nm; thus, it produces much higher amplitude PA signals than blood at this wavelength. Due to their small sizes and the liquid characteristics, the droplets flowed smoothly in blood vessels and provided significant PA contrast in the bloodstream. The resolution enhancement was demonstrated by continuously imaging the cortical layer of a mouse brain during droplet injection. The droplets that were flowing in vessels were localized, and their centers were used to construct an image of finer resolution than the acoustic diffraction limit. The superresolution PACT images outperformed the conventional PACT images in terms of feature sharpness and vessel resolution; the spatial resolution was improved by a factor of 6. Single droplets were also trackable in the brain at a depth of 4 mm, as demonstrated by imaging the mouse brain in a coronal plane, and the droplet flow directions and speeds were characterized in the deep brain.

Droplet-localization PACT can be improved further. First, dyed droplets with higher optical absorption would increase the spatial resolution due to the inverse relationship between the localization precision and the CNR of single droplets^24^. Thus, photoacoustically brighter dye at a longer wavelength (1.0–1.7 µm) would enable superresolution imaging of the vasculature in deeper tissues. Second, the sizes of the droplets are dispersed in this work, and many small droplets generated a background in the PACT images, thereby degrading the localization precision. This problem could be circumvented by generating mono-dispersed droplets using a microfluidic device^25^. Third, some droplets displayed moon-like shapes (see Supplementary Movie 2), which was probably due to their deviation from the focal plane of the transducers; thus, 2-D Gaussian fitting to these images produced a bias in their center estimation. An estimator with less bias is required for processing the images of these droplets. Fourth, the current imaging frame rate is 20 Hz, which is limited by the laser repetition rate. Although the droplets move slowly in terms of absolute speed (1.3–7.5 mm s^-1^), they move fast on a superresolution scale, i.e., the droplets displace 1/2 of the superresolution pixel width (25 µm/2) over a short time interval. For capturing the minute displacement accurately, high frame rates are required. Moreover, a high frame rate can effectively reduce the data acquisition time and facilitate the removal of the artifacts that resulted from the limited number of localized droplets. The technique that was developed in this work is expected to find wide applications in imaging blood vessels and monitoring targeted drug delivery in deep tissues.

## Materials and methods

### PACT system

The experimental setup for superresolution PACT is shown in Fig. 1. A piece of plastic film was used to seal the bottom of the full-ring ultrasonic transducer array, and water was poured in for ultrasonic coupling. The object to be imaged was either submerged in the water or placed just beneath the plastic film after it was lifted. A Ti:sapphire laser (LS-2145-LT-150, Symphotic Tii; 20-Hz pulse repetition rate; 12-ns pulse width) was used to excite PA waves. The laser beam was homogenized and expanded by an engineered diffuser (EDC10-A-1r, RPC Photonic), thereby resulting in a 2-cm-diameter illumination area on the object. The excited PA waves were detected by a 512-element full-ring ultrasonic transducer array (Imasonic Inc.; 50-mm ring radius; 5-MHz central frequency; more than 90% one-way bandwidth). Each element has a cylindrical focus (numerical aperture of 0.2; 20-mm element elevation size; 0.61-mm pitch; 0.1-mm interelement spacing). The PA signals were preamplified by a lab-made 512-channel preamplifier (26-dB gain) that was directly connected to the ultrasonic transducer array housing to reduce cable noise. The preamplified PA signals were digitized using four 128-channel data acquisition systems (SonixDAQs, Ultrasonix Medical ULC; 40-MHz sampling rate; 12-bit dynamic range) with a programmable amplification of up to 51 dB. The digitized data were initially stored in the onboard buffer and subsequently transferred to a computer for data processing.

### Dyed droplet preparation

In this work, a hydrophobic dye, namely, IR-780 iodide (425311, Sigma-Aldrich), was used as an optical contrast agent for superresolution PACT. A mixture of 67% (v/v) clove oil (C8392, Sigma-Aldrich) and 33% (v/v) peanut oil (P2144, Sigma-Aldrich) was used as the solvent, and it took 24–48 h to realize a maximum concentration of 2 mM. The oil mixture was prepared such that the final solution had a density that was close to that of water, which endowed the droplets with satisfactory stability in water. To generate droplet suspension for superresolution PACT, a mixture of 20 μL of the dye solution (2 mM) and 2 μL of surfactant (span®80, S6760-250ML, Sigma-Aldrich) was injected into 1 mL of distilled water and vibrated for 10 s using a mixer. The surfactant was used to prevent droplet coalescence. The resulting droplet suspension had a concentration of approximately 4 × 10^7^ mL^−1^.

### Animal surgery and preparation

In this work, 6- to 8-week-old female mice (Swiss Webster, Invigo) were used for in vivo imaging. To introduce the droplets into the brain, the left carotid artery of the mouse was cannulated with a polytetrafluoroethylene (PTFE) catheter, through which the droplet suspension was injected into the heart. Then, the droplets were pumped quickly by the heart into the brain through the right carotid artery. The cannulation procedure followed the protocol that was reported previously^26^. During the surgery, the mouse was anesthetized by 2% isoflurane at an air flow rate of 1 L min^-1^. A 1-cm longitudinal incision was made at the midline of the neck, and the omohyoid muscle was retracted to expose the left carotid artery. The vessel was clamped at the caudal end, and an incision was carefully made at the cranial end. A catheter was inserted toward the heart and secured by making a permanent suture ligature around the catheter and the vessel. During in vivo imaging, the mouse head was fixed by a nasal cone and a tooth bar. A rubber tube connected the nasal cone to an isoflurane vaporizer for delivering medical-grade air and anesthetic gas, as shown in Fig. 1b. Prior to brain imaging, the hair on the mouse head was removed by depilatory cream, and the scalp was cut to expose the entire cortical layer while keeping the skull intact. The surgical and imaging procedures followed the laboratory animal protocols that were approved by the Animal Studies Committee of Washington University in St. Louis.

### Data processing

The RF signals that were acquired from the 512-channel DAQ systems were jitter-corrected by using the PA signals from the surfaces of the ultrasonic transducer elements as the reference timings. Then, the initial pressure distribution in the object was retrieved by using the universal back-projection algorithm^20^, with a pixel size of 25 μm, which is 1/6 of the in-plane resolution (~150 μm) of the PACT system. The reconstructed bipolar image was converted into a unipolar image either by applying a Hessian-based Frangi vesselness filter^27^ for display or by ignoring the negative components for quantitative analysis.

To quantify the droplet flow speed in vessels, the PA amplitude profiles along the vessels were extracted, and the time-lapse profiles were organized to form images in the space-time domain. The 2-D Fourier transform was applied to these images, which mapped the lines that were of the same slope onto a single line through the origin in the spatiotemporal frequency domain, as shown in Supplementary Fig. S8. The location of this line in the quadrants indicated the flow direction, and its slope was a good estimate of the overall flow speed.

To track single droplets in vessels by PACT, the time-lapse images that were reconstructed from the RF data were denoised by applying a 2-D adaptive noise-removal filter^28^. Then, adjacent frames were subtracted to highlight flowing single droplets. To localize the single droplets, the differential grayscale images were converted into binary images by thresholding the pixel values at 1/4 of their maxima. The bright spots within a range of 16 to 64 pixels in the binary images were considered to be the regions that contained droplets. Any spots that had a roundness of less than 0.7 were discarded; hence, droplet clusters and artifacts were rejected. The centroids of the bright spots in the binary images were identified by using the image processing toolbox in MATLAB (Mathworks, Inc.), thereby coarsely locating the single droplets in the differential grayscale images. Then, for each droplet, a region of interest (ROI) that was centered at its centroid was isolated from the grayscale images. The ROI, which was of size 11 × 11 pixels, was fitted with a 2-D Gaussian function to realize more precise localization of the droplet. Each droplet was represented by a 2-D Gaussian-distributed spot that was located at its center with a radius that was equal to its localization uncertainty. By combining all the droplet images, an image with a spatial resolution that exceeds the acoustic diffraction limit was obtained. A pixel size of 5 μm was used in the superresolution image reconstruction.

## Supplementary information

Supplementary information

Movie 1

Movie 2

Movie 3

Movie 4
