# Supplementary material for: In vivo superresolution photoacoustic computed tomography by localization of single dyed droplets
Source: Light Sci Appl. 2019 Apr 3;8:36. doi: 10.1038/s41377-019-0147-9 (PMC6445830; doi:10.1038/s41377-019-0147-9)
Supplement: Supplementary file 1 — Supplementary information [file 41377_2019_147_MOESM1_ESM.docx]

***In vivo* superresolution photoacoustic computed tomography by localization of single dyes droplets: *Supplementary Figures***

Pengfei Zhang^1,2†^, Lei Li^3†^, Li Lin^3^, Junhui Shi^3^, and Lihong V. Wang^3*^

*^1^Optical Imaging Laboratory, Department of Biomedical Engineering, Washington University in St. Louis, St. Louis, Missouri 63130, USA*

*^2^Present address: School of Precision Instruments and Optoelectronics Engineering, Tianjin University, Tianjin, 300072, China*

^3^Caltech Optical Imaging Laboratory, Andrew and Peggy Cherng Department of Medical Engineering, Department of Electrical Engineering, California Institute of Technology, 1200 E. California Blvd., MC 138-78, Pasadena, CA 91125, USA

†These authors contributed equally to this work.

**Corresponding author: L.V.W. (*[*LVW@caltech.edu*](mailto:lhwang@biomed.wustl.edu)*)*


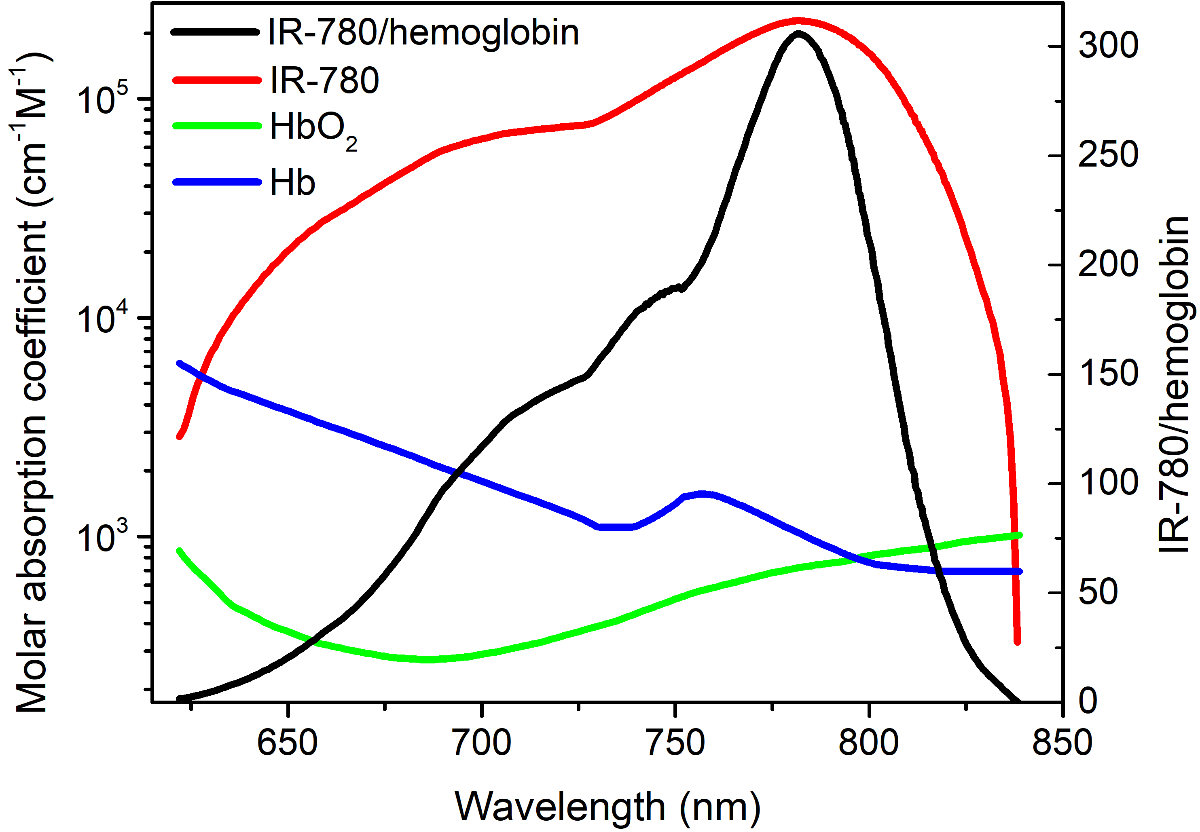


**Supplementary Figure S1 |** Absorption spectra of oxy-hemoglobin (HbO_2_), deoxy-hemoglobin (Hb) and IR-780 dye solution as well as the absorption coefficient ratio of IR-780 to hemoglobin (80% HbO_2_ and 20% Hb).


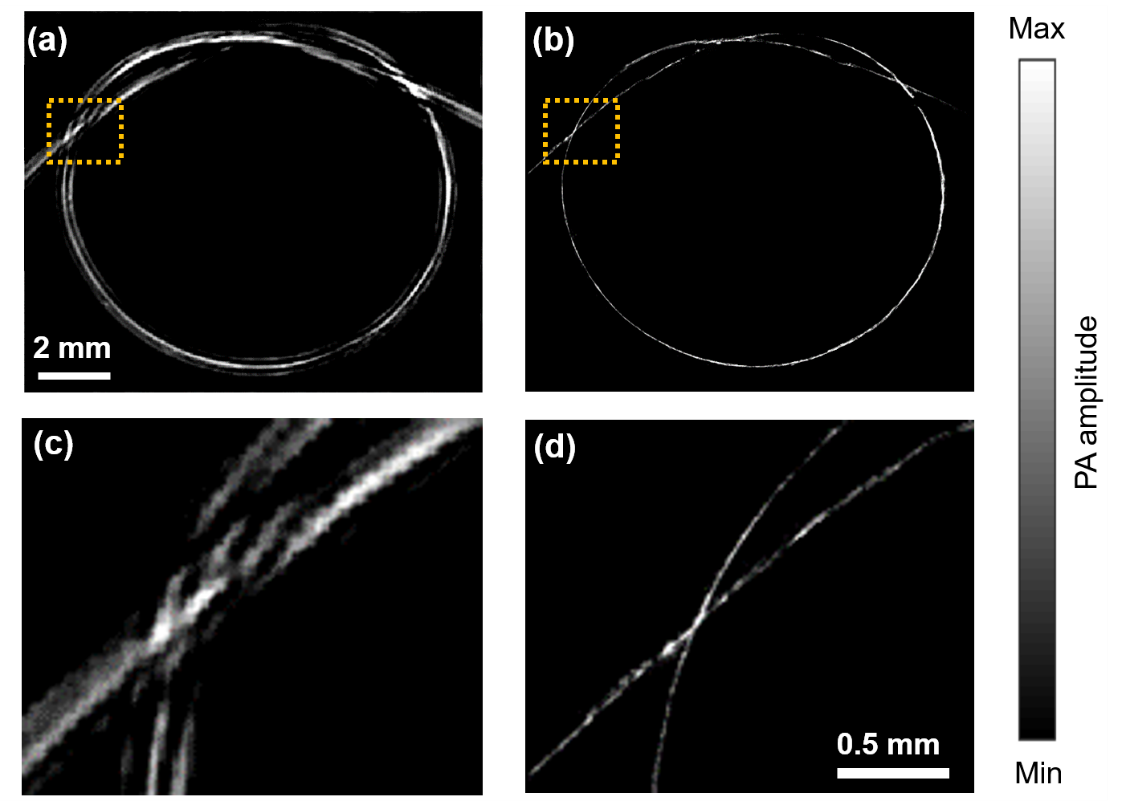


**Supplementary Figure S2 |** Imaging a knotted silicone capillary tube embedded in 3% agar gel by PACT. (a) A unipolar image of the tube acquired by conventional PACT. The negative amplitudes and the features outside the tube were removed. (b) A superresolution PACT image of the tube acquired by localizing single droplets. (c and d) Magnified images of the regions that are bounded by dotted rectangles in (a) and (b), respectively.


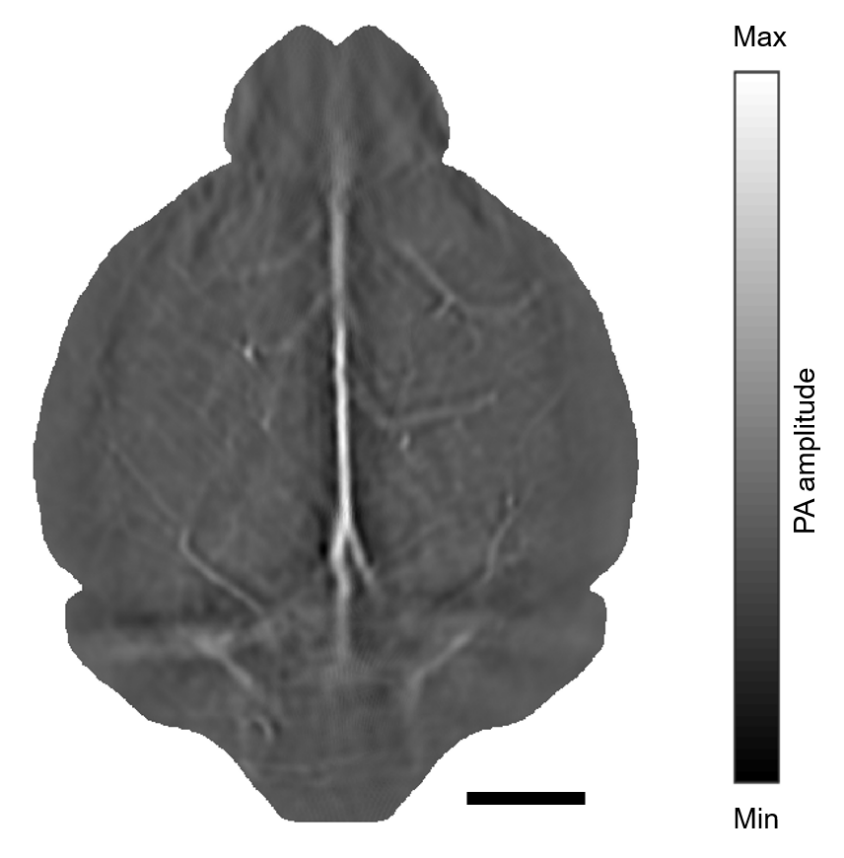


**Supplementary Figure S3 |** A reconstructed bipolar image of the cortical vasculature of a mouse brain acquired by conventional PACT. The scale bar is 2 mm.


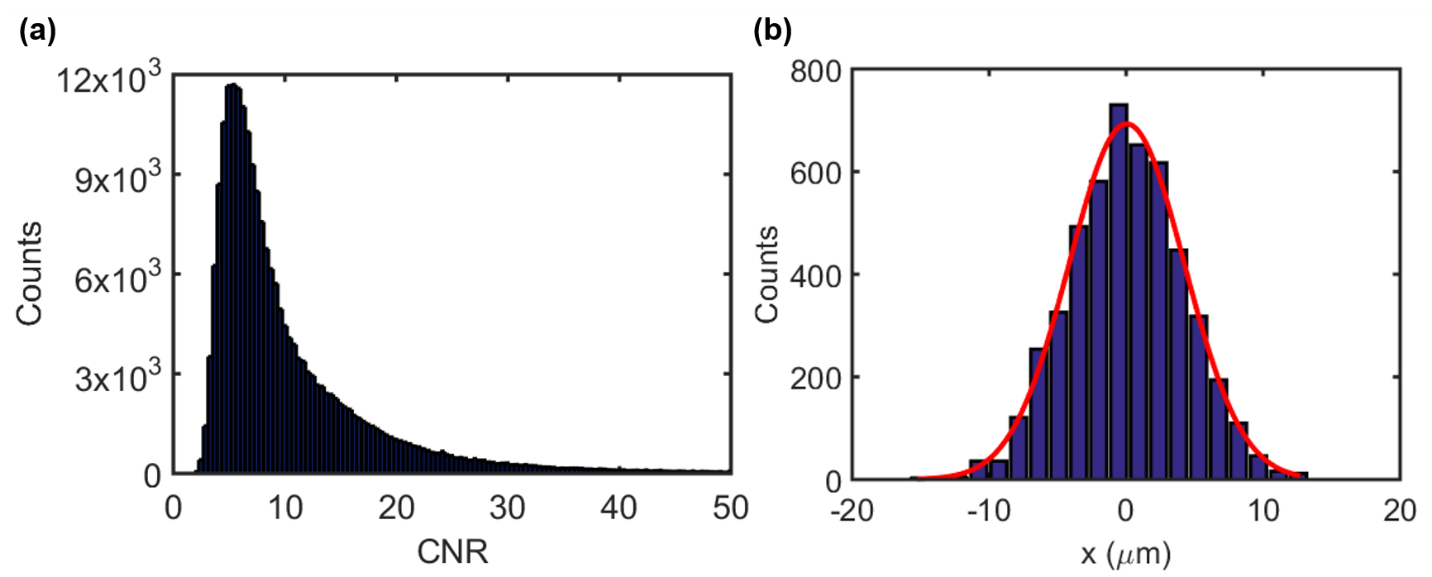


**Supplementary Figure S4 |** Droplet localization precision estimated from the PA contrast-to-noise ratios (CNRs) of the droplets and by numerical simulations. (a) A histogram of the experimental CNRs of the droplets. A total of ~220,000 droplets were counted, and a majority of the droplets exhibited CNRs of around 5. (b) A histogram of the localized positions of a droplet simulated with a CNR of 5. A total of 5000 simulations were made, and the droplet center was determined by a 2-D Gaussian fitting. The full width at half maximum (FWHM) of the histogram was determined to be 10 μm in (b). The simulations were performed based on the assumption that the image of a single droplet has a Gaussian distribution with both the peak amplitude and the radius close to the typical values determined in the experiments. Then Gaussian noise was added to give a CNR of 5.


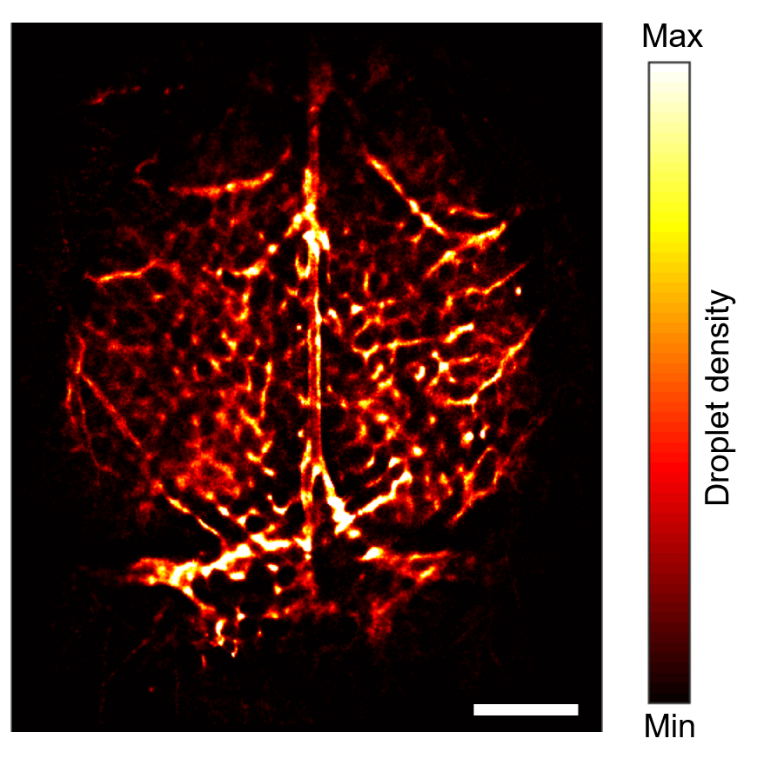


**Supplementary Figure S5 |** A superresolution image of the cortical vasculature of the mouse brain constructed from the centers of the localized droplets. The scale bar is 2 mm.


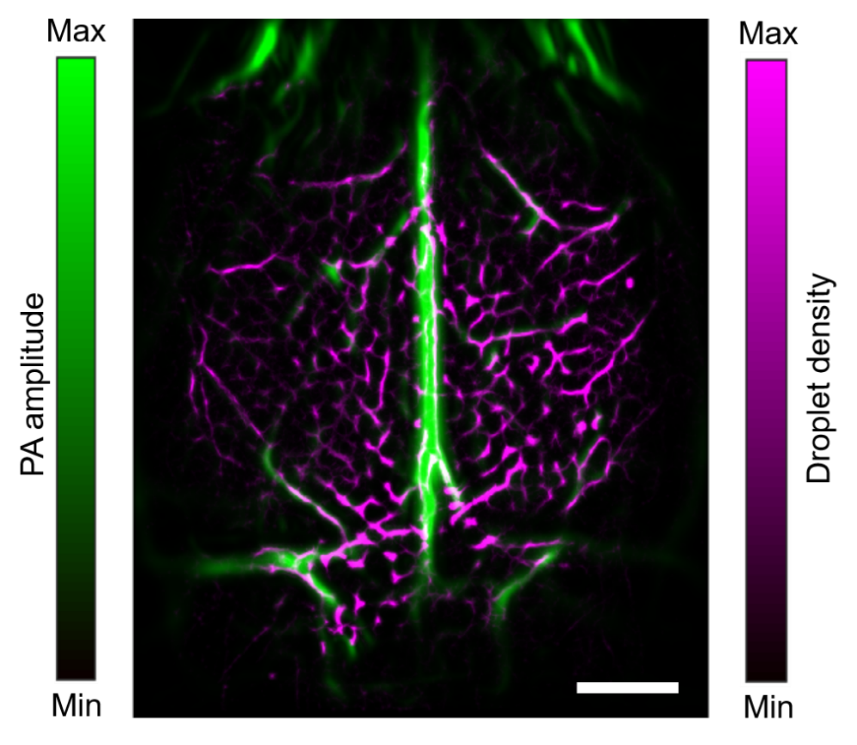


**Supplementary Figure S6 |** An overlay of the superresolution image (purple) and the conventional image (green). The scale bar is 2 mm.


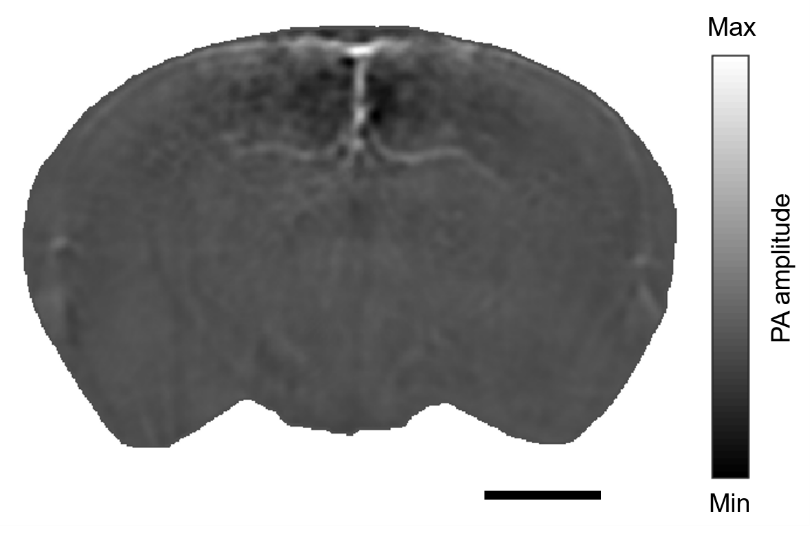


**Supplementary Figure S7 |** A reconstructed bipolar image of a mouse brain in the coronal plane (Bregma –1.0 mm) acquired by conventional PACT. The scale bar is 2 mm.


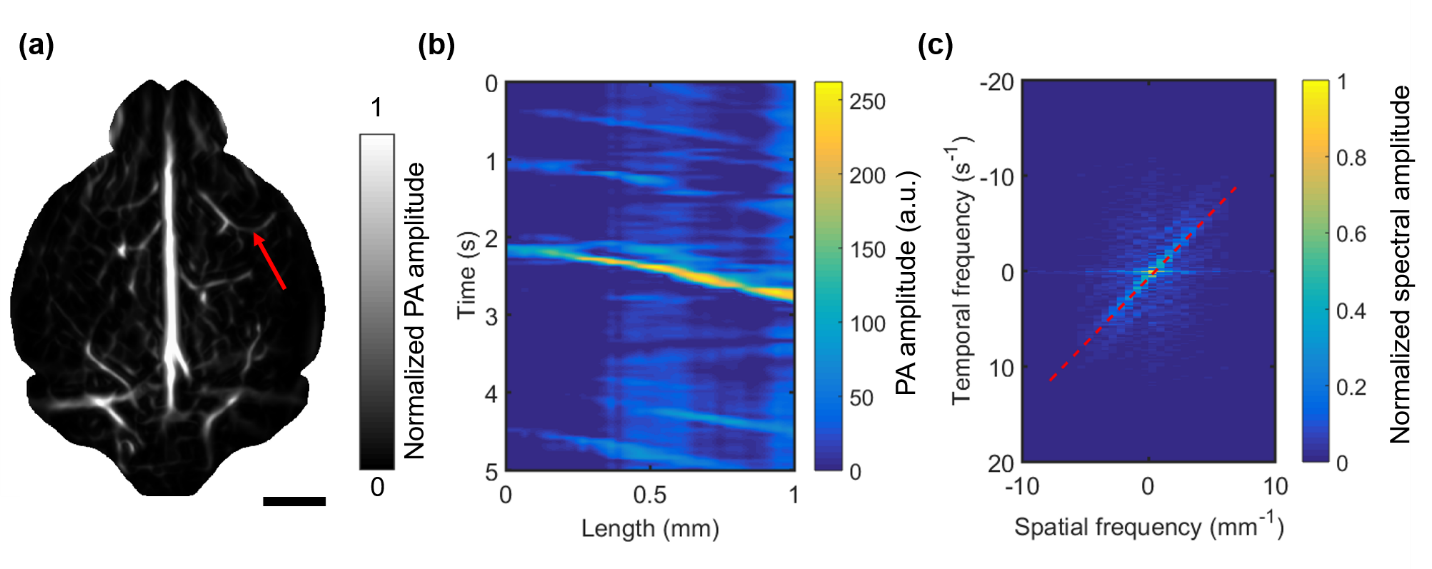


**Supplementary Figure S8 |** Quantification of droplet flow speed in vessels. (a) A unipolar image of the cortical vasculature of a mouse brain. The scale bar is 2 mm. (b) The time trace plot of each pixel along the vessel indicated by the red arrow in (a). (c) The 2-D Fourier transform of (b), which maps the lines that were of the same slope in the space-time domain onto a single line through the origin in the spatiotemporal frequency domain. The slope of this line was determined by a linear fitting, resulting in a flow speed of 1.3 mm s^-1^.
